# Suicide Risk Among Individuals Diagnosed With Cancer in the US, 2000-2016

**DOI:** 10.1001/jamanetworkopen.2022.51863

**Published:** 2023-01-20

**Authors:** Xin Hu, Jiemin Ma, Ahmedin Jemal, Jingxuan Zhao, Leticia Nogueira, Xu Ji, K. Robin Yabroff, Xuesong Han

**Affiliations:** 1 Department of Health Policy and Management, Emory University Rollins School of Public Health, Atlanta, Georgia; 2 Merck & Co Inc, Kenilworth, New Jersey; 3 Surveillance and Health Equity Science, American Cancer Society, Atlanta, Georgia; 4 Department of Pediatrics, Emory University School of Medicine, Atlanta, Georgia; 5 Aflac Cancer & Blood Disorders Center, Children's Healthcare of Atlanta, Atlanta, Georgia

## Abstract

**Importance:**

Individuals diagnosed with cancer have elevated suicide risks compared with the general population. National estimates of suicide risks among individuals with cancer are lacking in the US, and knowledge about risk factors is limited.

**Objective:**

To provide contemporary estimates of suicide risks associated with cancer and to identify sociodemographic and clinical factors associated with suicide risks among individuals diagnosed with cancer.

**Design, Setting, and Participants:**

A population-based cohort of individuals diagnosed with cancer from January 1, 2000, to December 31, 2016, from 43 states in the US were followed up through December 31, 2016. Standardized mortality ratios (SMRs) were calculated adjusting for attained age at death, sex, and race and ethnicity groups to compare suicide risks in the cancer cohort vs the general US population. Cox proportional hazards regression models were fitted to identify cancer-specific risk factors of suicide among the cancer cohort. Analyses were conducted from October 27, 2020, to May 13, 2022.

**Main Outcomes and Measures:**

The main outcomes were risk of suicide death compared with the general population, measured by the standardized mortality ratio; and risk of suicide death associated with sociodemographic and clinical factors among individuals with cancer.

**Exposure:**

Diagnosis of cancer.

**Results:**

Among a total of 16 771 397 individuals with cancer, 8 536 814 (50.9%) were 65 years or older at cancer diagnosis, 8 645 631 (51.5%) were male, 13 149 273 (78.4%) were non-Hispanic White, and 20 792 (0.1%) died from suicide. The overall SMR for suicide was 1.26 (95% CI, 1.24-1.28), with a decreasing trend (from an SMR of 1.67 [95% CI, 1.47-1.88] in 2000 to 1.16 [95% CI, 1.11-1.21] in 2016). Compared with the general population, elevated suicide risks were observed in the cancer cohort across all sociodemographic groups, with particularly high SMRs among Hispanic individuals (SMR, 1.48; 95% CI, 1.38-1.58), Medicaid-insured individuals (SMR, 1.72; 95% CI, 1.61-1.84), Medicare-insured individuals 64 years or younger (SMR, 1.94; 95% CI, 1.80-2.07), or uninsured individuals (SMR, 1.66; 95% CI, 1.53-1.80). Moreover, the highest SMR was observed in the first 6 months after the cancer diagnosis (SMR, 7.19; 95% CI, 6.97-7.41). Among individuals diagnosed with cancer, relatively higher suicide risks (ie, hazard ratios) were observed for cancer types with a poor prognosis and high symptom burden in the first 2 years after diagnosis, including cancers of oral cavity and pharynx, esophagus, stomach, brain and other nervous system, pancreas, and lung. After 2 years, individuals with cancers subject to long-term quality-of-life impairments, such as oral cavity and pharynx, leukemia, female breast, uterine, and bladder, had higher suicide risks.

**Conclusions and Relevance:**

In this cohort study of individuals with cancer, elevated suicide risks remained despite a decreasing trend during the past 2 decades. Suicide risks varied by sociodemographic and clinical factors. Timely symptom management and targeted psychosocial interventions are warranted for suicide prevention in individuals diagnosed with cancer.

## Key Points

**Question:**

What is the suicide risk associated with cancer in the US, and what are sociodemographic and clinical risk factors for suicide among individuals diagnosed with cancer?

**Findings:**

In this population-based cohort study of individuals diagnosed with cancer in 2000 to 2016 from 43 states, suicide risk was 26% higher compared with the general population, with insurance status and ethnicity contributing to the elevated risk. Higher suicide risks were seen among individuals with poor-prognosis cancers within 2 years of diagnosis and with cancers prone to long-term quality-of-life impairments after 2 years.

**Meaning:**

These findings suggest that timely symptom management and targeted psychosocial interventions are warranted for suicide prevention in individuals diagnosed with cancer.

## Teaser

This cohort study examines the sociodemographic and clinical factors associated with suicide risk among individuals diagnosed with cancer in the US.

## Introduction

Suicide is a leading cause of death worldwide, with more than 700 000 persons dying by suicide every year globally.^1^ In the US, more than 45 000 people died from suicide in 2020, corresponding to a rate of 14.0 per 100 000 person-years.^2^ Previous studies,^3,4,5,6,7,8,9^ mostly from Europe and North America, reported up to 11 times higher risks of suicide death among individuals diagnosed with cancer compared with the general population. Although cancer remains the main cause of death among individuals diagnosed with cancer, the elevated suicide risk is concerning and potentially preventable.

Previously reported risk factors for suicide among individuals with cancer included male sex, older age, advanced stage at diagnosis, and rural residence.^3,4,5,6,7^ However, estimates in the US have been limited to the Surveillance, Epidemiology, and End Results Program (SEER) registries from 13 states or fewer, and the studies were not able to examine the contribution of some factors that are unique within the US context to increased suicide risks, such as state of residence, insurance coverage, ethnicity, and county-level socioeconomic status. Unlike most developed countries, the US does not have a universal health care system, and health insurance coverage is a strong determinant of health care access and health outcomes.^10,11,12^ In the US, the federal Medicare program insures Americans 65 years or older and certain younger people with disabilities or specific conditions; most Americans younger than 65 years receive private health insurance through employers; and the federally aided, state-operated Medicaid program provides insurance coverage for low-income people, with income eligibility varying by state.^11^ Although the Patient Protection and Affordable Care Act significantly expanded insurance coverage in the US in the last decade, approximately 30 million Americans lacked health insurance coverage in 2020.^13^ In addition, race and ethnicity in the US are social constructs strongly associated with social determinants of health, exposures to environmental risk factors, and health disparities.^14^ Moreover, few studies examined temporal trends in suicide risk among individuals with cancer, although the suicide rate has increased markedly among the general population in the US during the past 2 decades.

To fill the knowledge gap, this study aims to provide estimates of suicide death risks and to examine sociodemographic and clinical characteristics associated with suicide death risks. We used comprehensive data from population-based cancer registries in 43 states in the US. Evidence from this study can better inform future efforts to improve psychosocial care and symptom management among individuals diagnosed with cancer.

## Methods

### Data

For this cohort study, we used the Cancer Incidence in North America (CiNA) survival data set compiled by the North American Association of Central Cancer Registries (NAACCR), which contained data from the Centers for Disease Control and Prevention National Program of Cancer Registries and the National Cancer Institute SEER registries and were certified by NAACCR’s high-quality data standards.^15^ We identified 16 954 604 individuals of all ages whose first primary malignant cancer was diagnosed between January 1, 2000, and December 31, 2016, from population-based state cancer registries in 43 states that agreed to participate in this study and provided data usable for survival analysis. The NAACCR survival data set did not include individuals with unknown age or sex or with cancer diagnoses reported on death certificate or autopsy only. We further excluded individuals alive with survival time missing (n = 11 109 [0.1%]) or unknown race or ethnicity (n = 172 098 [1.0%]). Eligible individuals were followed up through the date of last contact, death, or December 31, 2016, whichever came first. Mortality data for the US general population in 2000 to 2016 were acquired from the National Center for Health Statistics.^16^ The study was based on deidentified data and determined to be exempt from review by the NAACCR Institutional Review Board; therefore, no informed consent was required. This study followed the Strengthening the Reporting of Observational Studies in Epidemiology (STROBE) reporting guideline.

### Measures

For individuals who died during the study period, cause of death was identified using the *International Statistical Classification of Diseases and Related Health Problems, Tenth Revision* (*ICD-10*) codes. Suicide death was identified using codes U03 (suicide terrorism), X60-X84 (intentional self-harm), and Y87.0 (sequelae of intentional self-harm). Individual-level covariates were abstracted from medical records and included sex, age at diagnosis (in 5-year intervals), race and ethnicity (to adjust in standardization and to examine racial and ethnic disparities; categories included Hispanic; non-Hispanic American Indian, Alaska Native, Asian, or Pacific Islander; non-Hispanic Black; and non-Hispanic White), year of diagnosis, state of residence, SEER summary stage, and known primary payer at diagnosis (ie, insurance type), which was categorized as private, Medicaid, Medicare for individuals 64 years or younger, Medicare for individuals 65 years or older, Veterans Affairs (VA) or Indian Health Service (IHS)/Public Health Service (PHS) insurance, and uninsured (see eTable 1 in Supplement 1 for the availability of insurance information). County-level covariates from the American Community Surveys, including rurality and percentage of persons living below the poverty level, were available for individuals diagnosed in 2005 or later and were included in subgroup analyses for individuals diagnosed in 2005 to 2016.

### Statistical Analysis

Analyses were conducted from October 27, 2020, to May 13, 2022. Individuals’ sociodemographic and clinical characteristics were described for the entire cohort, those who died within the study period, and those who died of suicide. To estimate suicide risks associated with cancer and identify risk factors, we conducted 2 separate sets of analyses. First, standardized mortality ratios (SMRs), reflecting the risk of suicide among individuals with cancer relative to the general population, were calculated as the observed number of suicides divided by the expected number of suicides in the cancer cohort.^17^ The expected number of suicides was calculated by multiplying the sex-, age-, and race- and ethnicity-specific suicide rate during the study period (2000-2016) from the general population by total person-years of the corresponding sex, age, and race and ethnicity group of the cancer cohort. Of note, we used attained age at death for standardization, calculated from age at diagnosis and survival time in months in the cancer cohort. Because exact age at diagnosis was not available, we imputed individuals’ age at diagnosis assuming a uniform distribution within the 5-year age-at-diagnosis group. Sensitivity analyses that assumed the smallest age or the largest age within the 5-year age group showed similar results (eTable 2 in Supplement 1). Overall SMR and SMRs stratified by clinical characteristics and by sociodemographic characteristics, including year of death, were generated.

Second, to identify cancer-specific risk factors for suicide death among individuals with cancer, we estimated hazard ratios (HRs) using Cox proportional hazards regression while controlling for other causes of death as competing risks.^18^ Unlike the SMR, for which the suicide risk is interpreted relative to the general population, an HR indicates the suicide risk relative to the reference group among individuals with cancer. Covariates were selected based on a priori knowledge and included year of diagnosis, age at diagnosis, sex, race and ethnicity, state, primary payer at diagnosis, cancer stage, and cancer site as fixed effects. County-level rurality and poverty were included in subgroup analyses among those diagnosed in 2005 to 2016. After testing the proportional hazards assumption for each variable using visual examination of cumulative incidence function, we included age at diagnosis, cancer stage, and cancer site as time-dependent variables in the extended models, for which HRs for the first 2 years and after 2 years of follow-up were generated.^19,20^

## Results

### Sample Characteristics and Overall SMR

The analytical cancer cohort consisted of 16 771 397 individuals with 93 476 318 person-years during 2000 to 2016. Overall, 8 125 766 individuals (48.5%) were female and 8 645 631 (51.5%) were male; 588 206 (3.5%) were American Indian, Alaska Native, Asian, or Pacific Islander; 1 255 786 (7.5%) were Hispanic; 1 778 132 (10.6%) were non-Hispanic Black; 13 149 273 (78.4%) were non-Hispanic White; and 9 800 370 (80.8%) resided in metropolitan areas. Among those whose health insurance information was available, most had private insurance (4 914 979 [42.3%]) or Medicare for individuals 65 years or older (4 690 270 [40.4%]). The most common cancer sites were prostate (2 559 281 [15.3%]), female breast (2 500 492 [14.9%]), lung and bronchus (2 213 830 [13.2%]), and colon and rectum (1 611 161 [9.6%]); 1 898 812 (11.3%) had multiple cancer diagnoses by the end of follow-up. A total of 7 972 782 individuals (47.5%) died during the study period, with 20 792 (0.3%) suicide deaths. Among individuals with cancer who died by suicide, 9049 deaths (43.5%) occurred within 2 years of cancer diagnosis and 4188 (20.1%) occurred within 6 months of diagnosis. The overall SMR for suicide was 1.26 (95% CI, 1.24-1.28) (Table 1).

**Table 1.** Characteristics of Study Cohort and SMRs for Suicide

Characteristic	Individuals with cancer, No. (row %)			SMR (95% CI)^a^
	Total cancers (N = 16 771 397)	Total deaths (n = 7 972 782)	Total suicide deaths (n = 20 792)	
Overall	16 771 397 (100)	7 972 782 (47.5)	20 792 (0.3)	1.26 (1.24-1.28)
Year of death				
2000	149 464 (0.9)	149 464 (1.9)	247 (1.2)	1.67 (1.47-1.88)
2001	268 362 (1.6)	268 362 (3.4)	457 (2.2)	1.64 (1.49-1.79)
2002	326 604 (1.9)	326 604 (4.1)	600 (2.9)	1.51 (1.39-1.63)
2003	369 459 (2.2)	369 459 (4.6)	715 (3.4)	1.48 (1.37-1.59)
2004	395 881 (2.4)	395 881 (5.0)	792 (3.8)	1.37 (1.28-1.47)
2005	428 200 (2.6)	428 200 (5.4)	883 (4.2)	1.31 (1.22-1.39)
2006	450 335 (2.7)	450 335 (5.6)	946 (4.5)	1.24 (1.16-1.32)
2007	470 632 (2.8)	470 632 (5.9)	1144 (5.5)	1.31 (1.24-1.39)
2008	494 666 (2.9)	494 666 (6.2)	1174 (5.6)	1.18 (1.11-1.25)
2009	511 953 (3.1)	511 953 (6.4)	1397 (6.7)	1.29 (1.22-1.35)
2010	529 337 (3.2)	529 337 (6.6)	1367 (6.6)	1.15 (1.09-1.21)
2011	547 771 (3.3)	547 771 (6.9)	1562 (7.5)	1.21 (1.15-1.26)
2012	566 240 (3.4)	566 240 (7.1)	1585 (7.6)	1.13 (1.08-1.19)
2013	587 680 (3.5)	587 680 (7.4)	1809 (8.7)	1.19 (1.13-1.24)
2014	603 425 (3.6)	603 425 (7.6)	1927 (9.3)	1.17 (1.12-1.23)
2015	629 898 (3.8)	629 898 (7.9)	2093 (10.1)	1.21 (1.16-1.27)
2016	642 875 (3.8)	642 875 (8.1)	2094 (10.1)	1.16 (1.11-1.21)
Alive	8 798 615 (52.5)	NA	NA	NA
Attained age at death, y				
0-24	41 651 (0.2)	41 651 (0.5)	106 (0.5)	1.05 (0.85-1.25)
25-39	109 513 (0.7)	109 513 (1.4)	557 (2.7)	1.06 (0.85-1.25)
40-49	342 521 (2.0)	342 521 (4.3)	1302 (6.3)	1.09 (1.03-1.15)
50-54	405 283 (2.4)	405 283 (5.1)	1411 (6.8)	1.13 (1.07-1.19)
55-59	607 509 (3.6)	607 509 (7.6)	1989 (9.6)	1.17 (1.12-1.22)
60-64	791 876 (4.7)	791 876 (9.9)	2461 (11.8)	1.31 (1.26-1.36)
65-69	944 816 (5.6)	944 816 (11.9)	2866 (13.8)	1.44 (1.39-1.50)
70-74	1 058 651 (6.3)	1 058 651 (13.3)	2926 (14.1)	1.38 (1.33-1.43)
75-79	1 136 998 (6.8)	1 136 998 (14.3)	2965 (14.3)	1.39 (1.34-1.44)
80-84	1 103 302 (6.6)	1 103 302 (13.8)	2345 (11.3)	1.26 (1.21-1.31)
≥85	1 430 662 (8.5)	1 430 662 (17.9)	1864 (9.0)	1.07 (1.03-1.12)
Alive	8 798 615 (52.5)	NA	NA	NA
Sex				
Male	8 645 631 (51.5)	4 329 718 (54.3)	17 572 (84.5)	1.29 (1.27-1.30)
Female	8 125 766 (48.5)	3 643 064 (45.7)	3220 (15.5)	1.14 (1.10-1.18)
Race and ethnicity				
Hispanic	1 255 786 (7.5)	505 830 (6.3)	785 (3.8)	1.48 (1.38-1.58)
Non-Hispanic				
AIANAPI	588 206 (3.5)	245 128 (3.1)	475 (2.3)	1.79 (1.63-1.95)
Black	1 778 132 (10.6)	896 830 (11.2)	632 (3.0)	1.15 (1.06-1.24)
White	13 149 273 (78.4)	6 324 994 (79.3)	18 900 (90.9)	1.25 (1.23-1.27)
Region				
Northeast	2 212 373 (13.2)	1 044 950 (13.1)	2014 (9.7)	1.25 (1.20-1.30)
Midwest	3 128 127 (18.7)	1 507 915 (18.9)	3529 (17.0)	1.25 (1.21-1.30)
South	7 279 094 (43.4)	3 540 101 (44.4)	9222 (44.4)	1.21 (1.18-1.23)
West	4 151 803 (24.8)	1 879 816 (23.6)	6027 (29.0)	1.18 (1.15-1.21)
Insurance type^b^				
Private	4 914 979 (29.3)	1 523 193 (19.1)	5450 (26.2)	1.08 (1.05-1.11)
Medicaid	780 220 (4.7)	407 944 (5.1)	851 (4.1)	1.72 (1.61-1.84)
Medicare ≤64 y of age	540 921 (3.2)	261 066 (3.3)	809 (3.9)	1.94 (1.80-2.07)
Medicare ≥65 y of age	4 690 270 (28.0)	2 742 090 (34.4)	5790 (27.8)	1.42 (1.38-1.46)
VA or IHS/PHS	233 349 (1.4)	120 746 (1.5)	511 (2.5)	1.89 (1.72-2.05)
Uninsured	463 664 (2.8)	228 741 (2.9)	595 (2.9)	1.66 (1.53-1.80)
Unknown or missing	5 147 994 (30.7)	2 689 002 (33.7)	6786 (32.6)	1.17 (1.14-1.20)
County-level rurality^c^				
Metropolitan	9 800 370 (80.8)	3 935 950 (79.2)	9800 (77.9)	1.29 (1.27-1.32)
Nonmetropolitan				
Urban	1 823 072 (15.0)	824 569 (16.6)	2233 (17.8)	1.34 (1.28-1.40)
Rural	243 729 (2.0)	113 206 (2.3)	321 (2.6)	1.39 (1.24-1.54)
Unknown or missing	265 043 (2.2)	94 081 (1.9)	219 (1.7)	NA
County-level poverty, %^c^				
<5.0	601 585 (5.0)	208 402 (4.2)	493 (3.9)	1.24 (1.13-1.35)
5.0-9.99	3 701 312 (30.5)	1 430 150 (28.8)	3704 (29.5)	1.42 (1.38-1.47)
10.0-19.99	6 684 792 (55.1)	2 828 252 (56.9)	7306 (58.1)	1.35 (1.32-1.38)
≥20.0	879 482 (7.2)	406 921 (8.2)	851 (6.8)	1.35 (1.26-1.44)
Unknown	265 043 (2.2)	94 081 (1.9)	219 (1.7)	NA
Stage				
In situ or local	7 927 770 (47.3)	2 307 856 (28.9)	10 787 (51.9)	1.04 (1.02-1.06)
Regional	3 605 880 (21.5)	1 734 455 (21.8)	4397 (21.1)	1.46 (1.41-1.50)
Distant	3 833 893 (22.9)	2 932 044 (36.8)	3794 (18.2)	1.90 (1.83-1.96)
Not applicable, unknown, or missing	1 403 854 (8.4)	998 427 (12.5)	1814 (8.7)	1.62 (1.54-1.69)
Number of primary cancers				
1	14 872 585 (88.7)	7 074 007 (88.7)	18 631 (89.6)	1.35 (1.34-1.37)
≥2	1 898 812 (11.3)	898 775 (11.3)	2161 (10.4)	0.79 (0.76-0.82)
Time since diagnosis at death				
0-5 mo	NA	2 633 423 (33.0)	4188 (20.1)	7.19 (6.97-7.41)
6-11 mo	NA	1 080 065 (13.5)	1987 (9.6)	5.60 (5.35-5.84)
12-23 mo	NA	1 204 531 (15.1)	2874 (13.8)	4.18 (4.03-4.33)
24-35 mo	NA	689 630 (8.6)	2145 (10.3)	3.09 (2.96-3.22)
3-4 y	NA	852 473 (10.7)	3083 (14.8)	1.98 (1.91-2.05)
5-9 y	NA	1 086 184 (13.6)	4688 (22.5)	0.94 (0.91-0.97)
≥10 y	NA	426 476 (5.3)	1827 (8.8)	0.24 (0.23-0.25)
Primary site				
Female breast^d^	2 500 492 (14.9)	667 170 (8.4)	1187 (5.7)	1.03 (0.97-1.09)
Prostate	2 559 281 (15.3)	730 459 (9.2)	5083 (24.4)	0.91 (0.89-0.94)
Colon and rectum	1 611 161 (9.6)	851 611 (10.7)	1992 (9.6)	1.25 (1.20-1.31)
Lung and bronchus	2 213 830 (13.2)	1 852 179 (23.2)	2462 (11.8)	2.34 (2.25-2.44)
Uterine corpus	1 008 235 (6.0)	394 540 (4.9)	431 (2.1)	1.11 (1.01-1.22)
Oral cavity and pharynx	409 033 (2.4)	195 718 (2.5)	1157 (5.6)	2.42 (2.28-2.56)
Kidney and renal pelvis	549 636 (3.3)	221 023 (2.8)	673 (3.2)	1.14 (1.05-1.23)
Melanoma	674 205 (4.0)	162 744 (2.0)	1017 (4.9)	1.03 (0.97-1.09)
Non-Hodgkin lymphoma	693 316 (4.1)	314 827 (3.9)	826 (4.0)	1.17 (1.09-1.25)
Thyroid	439 331 (2.6)	38 511 (0.5)	306 (1.5)	0.82 (0.73-0.91)
Pancreas	425 278 (2.5)	375 122 (4.7)	354 (1.7)	2.50 (2.24-2.76)
Liver and intrahepatic bile duct	268 348 (1.6)	213 563 (2.7)	189 (0.9)	1.59 (1.36-1.82)
Bladder	700 303 (4.2)	329 822 (4.1)	1396 (6.7)	1.27 (1.21-1.34)
Esophagus	170 794 (1.0)	139 525 (1.8)	365 (1.8)	3.15 (2.82-3.47)
Leukemia	472 297 (2.8)	246 433 (3.1)	476 (2.3)	1.17 (1.06-1.27)
Brain and other nervous system	253 327 (1.5)	169 642 (2.1)	251 (1.2)	1.67 (1.47-1.88)
Stomach	239 489 (1.4)	177 313 (2.2)	317 (1.5)	2.32 (2.07-2.58)
Other	1 583 041 (9.4)	892 580 (11.2)	2310 (11.1)	1.62 (1.55-1.68)

Abbreviations: AIANAPI, American Indian, Alaska Native, Asian, or Pacific Islander; IHS, Indian Health Service; NA, not applicable; PHS, Public Health Service; SMR, standardized mortality ratio; VA, Veterans Affairs.

^a^ The SMRs are standardized by attained age at death, sex, and race and ethnicity. The SMRs for year of death, region, county-level rurality, and county-level poverty subcategories were also standardized by year of death, region, county-level rurality, and county-level poverty, respectively.

^b^ Private insurance included private fee-for-service and managed care, TRICARE, and military insurance; Medicaid included traditional Medicaid, managed care Medicaid, and other not specified Medicaid without Medicare; and Medicare included traditional fee-for-service Medicare, Medicare administered through a managed care plan, Medicare with supplemental coverage, and Medicare with Medicaid eligibility.

^c^ County-level data only available in 2005 and after (N = 11 867 171). County-level rurality was coded with Rural-Urban Continuum Codes developed by the US Department of Agriculture. County-level poverty was coded with percentage of persons below the poverty level data from the American Community Survey, 2007-2011.

^d^ Male breast cancer was grouped into the “other” category.

### Suicide Risks of Cancer Cohort vs General Population by Sociodemographic Characteristics

**Figure 1.**
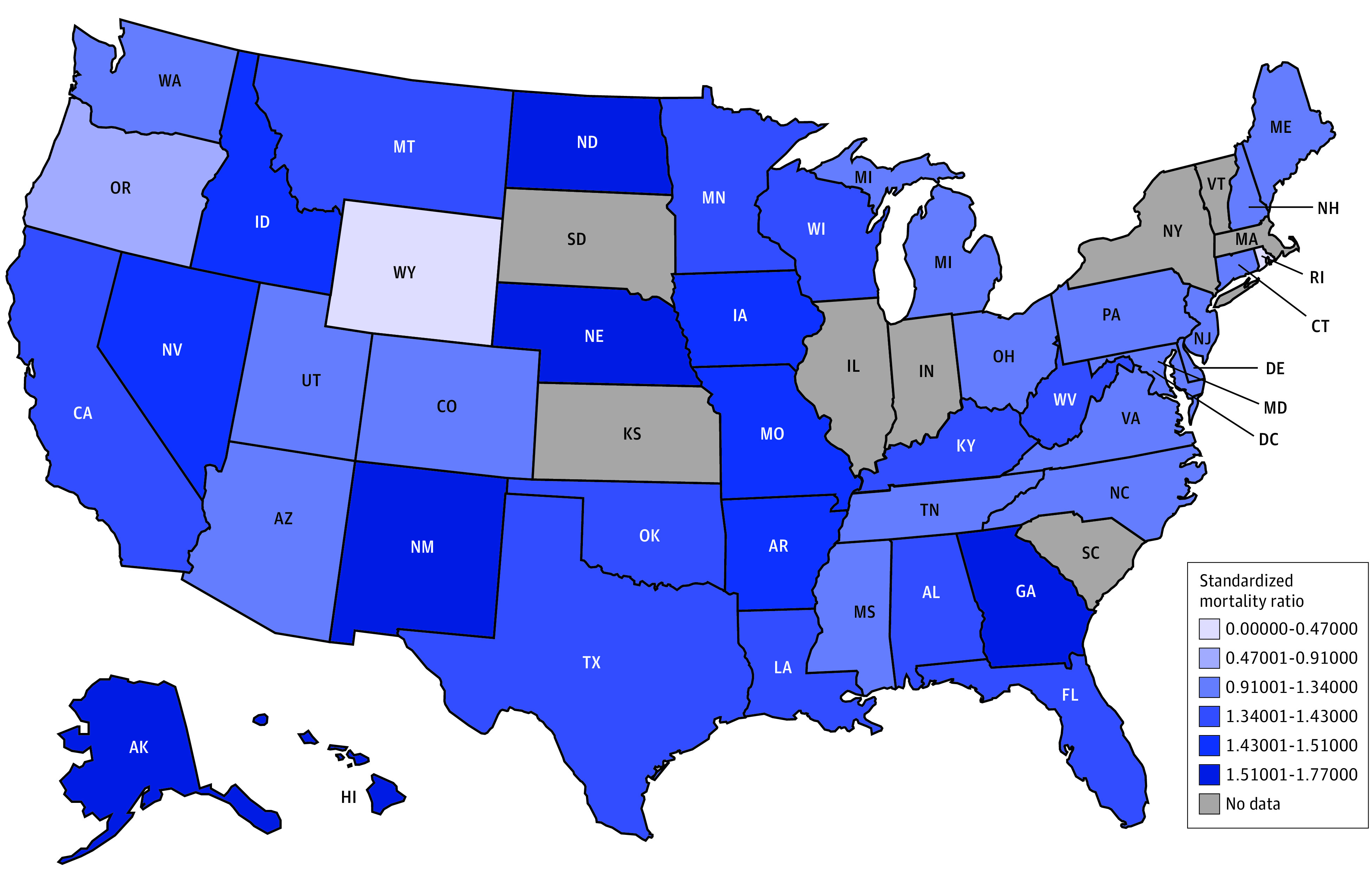
Standardized Mortality Ratios by State, 2000-2016 Standardized mortality ratios are standardized by age group only because of the small population in certain states.

During the study period, the age-standardized SMRs by state ranged from 0.47 (95% CI, 0.28-0.65) in Wyoming to 1.77 (95% CI, 1.39-2.14) in Alaska (Figure 1). Individuals with cancer in Alaska, North Dakota, Nebraska, and New Mexico had the highest SMRs for suicide compared with the general US population.

**Figure 2.**
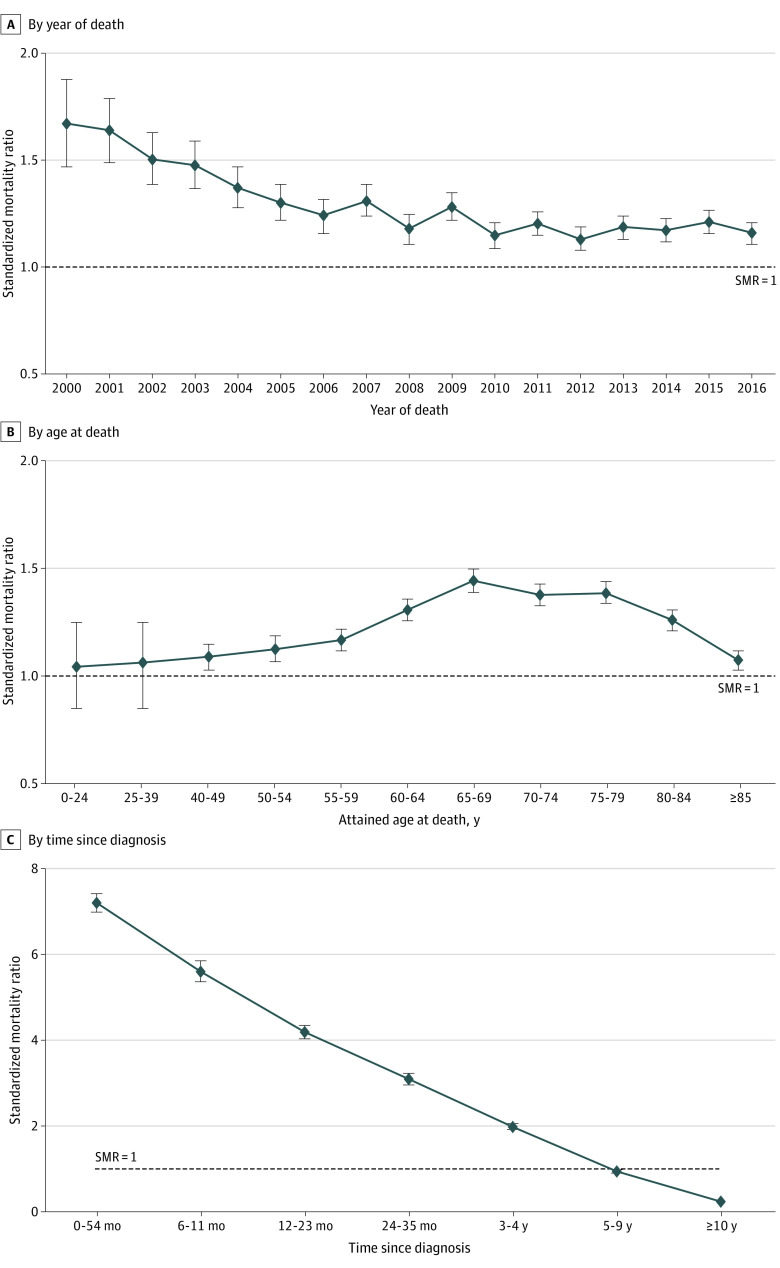
Standardized Mortality Ratios by Year of Death, Age at Death, and Time Since Diagnosis, 2000-2016 Standardized mortality ratios are standardized by age at death, sex, and race and ethnicity. The standardized mortality ratio for year at death was also standardized by year.

The SMRs by year of death were plotted to examine the time trend in suicide risks associated with cancer (Figure 2A). Although individuals diagnosed with cancer had consistently higher suicide risks during the entire study period, elevated risks decreased from 1.67 (95% CI, 1.47-1.88) in 2000 to 1.15 (95% CI, 1.09-1.21) in 2010 and then leveled off with small fluctuations, with an SMR of 1.16 (95% CI, 1.11-1.21) in 2016.

Individuals diagnosed with cancer had significantly higher suicide risks than the general population in all attained age groups older than 40 years at death. There was an increasing trend of SMRs by attained age up to 65 to 69 years (SMR, 1.44; 95% CI, 1.39-1.50) followed by a decreasing trend among age groups older than 69 years (Table 1, Figure 2B).

Compared with their counterparts among the general population, we found higher SMRs among individuals diagnosed with cancer who were male (SMR, 1.29; 95% CI, 1.27-1.30), Hispanic (SMR, 1.48; 95% CI, 1.38-1.58), American Indian, Alaska Native, Asian, or Pacific Islander (SMR, 1.79; 95% CI, 1.63-1.95), insured with Medicaid (SMR, 1.72; 95% CI, 1.61-1.84), insured with Medicare for those 64 years or younger (SMR, 1.94; 95% CI, 1.80-2.07), insured by the VA or IHS/PHS (SMR, 1.89; 95% CI, 1.72-2.05), uninsured (SMR, 1.66; 95% CI, 1.53-1.80), and residing in rural areas (SMR, 1.39; 95% CI, 1.24-1.54) (Table 1).

### Suicide Risks of Cancer Cohort vs General Population by Clinical Characteristics

Suicide risk in the cancer cohort was substantially higher than the general population within the first 6 months of diagnosis (SMR, 7.19; 95% CI, 6.97-7.41) and decreased with longer time since cancer diagnosis but remained higher than the general population until 5 years after diagnosis (SMR, 0.94; 95% CI, 0.91-0.97 for 5 to 9 years since diagnosis) (Table 1, Figure 2C).

Suicide risks were higher among individuals diagnosed with cancer compared with the general population across all cancer stages, with the highest SMR for distant stage (SMR, 1.90; 95% CI, 1.83-1.96) followed by regional stage (SMR, 1.46; 95% CI, 1.41-1.50) and in situ or local stages (SMR, 1.04; 95% CI, 1.02-1.06) (Table 1). Across cancer sites, the highest SMRs were observed among individuals diagnosed with cancers of lung and bronchus (SMR, 2.34; 95% CI, 2.25-2.44), oral cavity and pharynx (SMR, 2.42; 95% CI, 2.28-2.56), pancreas (SMR, 2.50; 95% CI, 2.24-2.76), esophagus (SMR, 3.15; 95% CI, 2.82-3.47), and stomach (SMR, 2.32; 95% CI, 2.07-2.58). In contrast, individuals diagnosed with prostate (SMR, 0.91; 95% CI, 0.89-0.94) and thyroid (SMR, 0.82; 95% CI, 0.73-0.91) cancers showed lower suicide risks than the general population.

### Risk Factors for Suicide Among Individuals Diagnosed With Cancer

The Cox proportional hazards regressions showed that, compared with individuals diagnosed with cancer in 2000, individuals diagnosed in recent years had significantly lower suicide risks (HRs <1.0 after 2009) (eFigure A in Supplement 1). Compared with individuals residing in the most populous state (California), individuals residing in New Mexico (HR, 1.68; 95% CI, 1.48-1.91), Nevada (HR, 1.59; 95% CI, 1.43-1.78), and Alaska (HR, 1.47; 95% CI, 1.19-1.83) had the highest suicide risks (eFigure B in Supplement 1). Individuals who were male (vs female), non-Hispanic White (vs other racial or ethnic minorities), and living in nonmetropolitan (vs metropolitan) counties and counties of high (vs low) poverty also had higher suicide risks (Table 2; eTable 3 in Supplement 1). Compared with privately insured individuals, individuals 64 years or younger insured with Medicare (HR, 1.48; 95% CI, 1.37-1.60), VA or IHS/PHS (HR, 1.34; 95% CI, 1.22-1.47), Medicaid (HR, 1.23; 95% CI, 1.14-1.33), and uninsured (HR, 1.27; 95% CI, 1.16-1.38) had higher suicide risks (Table 2).

**Table 2.** Risk of Suicide Associated With No Time-Interaction Characteristics Among the Cancer Cohort, 2000-2016^a^

Characteristic	Univariable regression (n = 16 771 397)		Multivariable regression (n = 16 771 397)	
	HR (95% CI)	*P* value	HR (95% CI)	*P* value
Sex				
Female	1 [Reference]	NA	1 [Reference]	NA
Male	5.06 (4.87-5.25)	<.001	5.52 (5.21-5.85)	<.001
Race and ethnicity				
Hispanic	0.46 (0.43-0.50)	<.001	0.43 (0.40-0.47)	<.001
Non-Hispanic				
AIANAPI	0.60 (0.55-0.66)	<.001	0.60 (0.54-0.66)	<.001
Black	0.25 (0.23-0.27)	<.001	0.25 (0.23-0.28)	<.001
White	1 [Reference]	NA	1 [Reference]	NA
Insurance type^b^				
Private	1 [Reference]	NA	1 [Reference]	NA
Medicaid	0.99 (0.92-1.06)	.78	1.23 (1.14-1.33)	<.001
Medicare				
≤64 y of age	1.38 (1.29-1.49)	<.001	1.48 (1.37-1.60)	<.001
≥65 y of age	1.12 (1.08-1.16)	<.001	1.14 (1.09-1.20)	<.001
VA or IHS/PHS	1.99 (1.82-2.18)	<.001	1.34 (1.22-1.47)	<.001
Uninsured	1.14 (1.04-1.24)	.003	1.27 (1.16-1.38)	<.001
Unknown or missing	1.07 (1.03-1.11)	<.001	1.06 (1.02-1.11)	.004
County-level rurality^c^				
Metropolitan	1 [Reference]	NA	1 [Reference]	NA
Nonmetropolitan				
Urban	1.22 (1.16-1.27)	<.001	1.09 (1.03-1.14)	.001
Rural	1.31 (1.17-1.46)	<.001	1.14 (1.02-1.28)	.03
County-level poverty, %^c^				
<5.0	1 [Reference]	NA	1 [Reference]	NA
5.0-9.99	1.22 (1.11-1.34)	<.001	1.07 (0.97-1.18)	.17
10.0-19.99	1.33 (1.21-1.45)	<.001	1.13 (1.02-1.25)	.02
≥20.0	1.17 (1.05-1.31)	.005	1.12 (0.99-1.27)	.07

Abbreviations: AIANAPI, American Indian, Alaska Native, Asian, or Pacific Islander; HR, hazard ratio; IHS, Indian Health Service; NA, not applicable; PHS, Public Health Service; VA, Veterans Affairs.

^a^ Cox proportional hazards regression models controlled for other cause of death as competing risks. Models also adjusted for characteristics in Table 3 and age at diagnosis, North American Association of Central Cancer Registries, and year of diagnosis, with results shown in the eFigure in Supplement 1.

^b^ Private insurance included private fee-for-service and managed care, TRICARE, and military insurance; Medicaid included traditional Medicaid, managed care Medicaid, and other not specified Medicaid without Medicare; and Medicare included traditional fee-for-service Medicare, Medicare administered through a managed care plan, Medicare with supplemental coverage, and Medicare with Medicaid eligibility.

^c^ Estimates for county-level rurality and poverty came from separate models among individuals diagnosed with cancer in 2005 to 2016 (n = 11 867 171) because county-level data were only available in 2005 and after. Estimates for the full model among this subcohort are provided in eTable 3 in Supplement 1. County-level rurality was coded with Rural-Urban Continuum Codes developed by the US Department of Agriculture. County-level poverty was coded with percentage of persons below the poverty level data from the American Community Survey, 2007-2011.

Suicide risks associated with age at diagnosis, cancer stage, and cancer site were time dependent. Specifically, within the first 2 years of cancer diagnosis, older age at diagnosis and more advanced cancer stage at diagnosis were associated with higher suicide risks; however, after 2 years of diagnosis, age of 25 to 49 years at diagnosis and in situ or local stage at diagnosis were associated with higher suicide risks (Table 2; eFigure C in Supplement 1). Using colorectal cancer (prevalent in both males and females) as the reference group, higher suicide risks were seen for the following cancer sites during the first 2 years of diagnosis: oral cavity and pharynx (HR, 2.07; 95% CI, 1.87-2.29), esophagus (HR, 2.13; 95% CI, 1.88-2.41), stomach (HR, 1.70; 95% CI, 1.48-1.94), brain and other nervous system (HR, 1.38; 95% CI, 1.18-1.61), lung and bronchus (HR, 1.31; 95% CI, 1.21-1.42), and pancreas (HR, 1.27; 95% CI, 1.12-1.43), whereas the lower suicide risks were seen for prostate (HR, 0.64; 95% CI, 0.59-0.70), leukemia (HR, 0.73; 95% CI, 0.63-0.85), liver and intrahepatic bile duct (HR, 0.78; 95% CI, 0.66-0.93), and melanoma (HR, 0.84; 95% CI, 0.74-0.95). In 2 or more years of follow-up after diagnosis, individuals with oral cavity and pharynx cancer still had the highest suicide risk (HR, 1.51; 95% CI, 1.36-1.68) followed by leukemia (HR, 1.49; 95% CI, 1.25-1.77), female breast cancer (HR, 1.24; 95% CI, 1.11-1.38), uterine cancer (HR, 1.17; 95% CI, 1.01-1.36), and bladder cancer (HR, 1.13; 95% CI, 1.03-1.24), whereas pancreas, brain and other nervous system, lung and bronchus, esophagus, stomach, and kidney and renal pelvis cancers showed relatively lower suicide risks compared with colorectal cancer (Table 3).

**Table 3.** Risk of Suicide Associated With Time-Interaction Characteristics Among the Cancer Cohort, 2000-2016^a^

Characteristic	Univariable regression (n = 16 771 397)				Multivariable regression (n = 16 771 397)			
	Within 2 y		After 2 y		Within 2 y		After 2 y	
	HR (95% CI)	*P* value	HR (95% CI)	*P* value	HR (95% CI)	*P* value	HR (95% CI)	*P* value
Stage								
In situ or local	1 [Reference]	NA	1 [Reference]	NA	1 [Reference]	NA	1 [Reference]	NA
Regional	1.41 (1.34-1.48)	<.001	0.68 (0.65-0.71)	<.001	1.29 (1.22-1.36)	<.001	0.85 (0.81-0.90)	<.001
Distant	1.63 (1.55-1.71)	<.001	0.33 (0.31-0.36)	<.001	1.32 (1.25-1.41)	<.001	0.41 (0.38-0.45)	<.001
Not applicable, unknown, or missing	1.78 (1.68-1.89)	<.001	0.54 (0.50-0.59)	<.001	1.39 (1.30-1.49)	<.001	0.66 (0.61-0.73)	<.001
Primary site								
Colon and rectum	1 [Reference]	NA	1 [Reference]	NA	1 [Reference]	NA	1 [Reference]	NA
Female breast^b^	0.28 (0.25-0.32)	<.001	0.47 (0.42-0.51)	<.001	1.06 (0.93-1.20)	.40	1.24 (1.11-1.38)	<.001
Prostate	0.95 (0.87-1.03)	.20	2.05 (1.91-2.19)	<.001	0.64 (0.59-0.70)	<.001	1.09 (1.01-1.17)	.02
Lung and bronchus	1.54 (1.43-1.66)	<.001	0.43 (0.38-0.48)	<.001	1.31 (1.21-1.42)	<.001	0.53 (0.47-0.60)	<.001
Uterine corpus	0.32 (0.27-0.37)	<.001	0.39 (0.34-0.45)	<.001	1.18 (1.00-1.39)	.05	1.17 (1.01-1.36)	.04
Oral cavity and pharynx	2.65 (2.40-2.93)	<.001	2.19 (1.98-2.43)	<.001	2.07 (1.87-2.29)	<.001	1.51 (1.36-1.68)	<.001
Kidney and renal pelvis	1.00 (0.89-1.13)	.98	1.10 (0.97-1.24)	.14	0.99 (0.87-1.12)	.84	0.86 (0.76-0.97)	.01
Melanoma	0.86 (0.76-0.97)	.02	1.62 (1.47-1.78)	<.001	0.84 (0.74-0.95)	.006	1.05 (0.95-1.16)	.33
Non-Hodgkin lymphoma	0.97 (0.87-1.09)	.65	1.00 (0.90-1.12)	.95	0.93 (0.82-1.04)	.19	1.12 (1.00-1.25)	.06
Thyroid	0.43 (0.35-0.52)	<.001	0.79 (0.68-0.92)	.002	0.90 (0.74-1.10)	.31	0.91 (0.78-1.06)	.21
Pancreas	1.35 (1.20-1.52)	<.001	0.17 (0.11-0.28)	<.001	1.27 (1.12-1.43)	<.001	0.25 (0.15-0.40)	<.001
Liver and intrahepatic bile duct	0.88 (0.74-1.05)	.15	0.38 (0.27-0.54)	<.001	0.78 (0.66-0.93)	.006	0.32 (0.23-0.46)	<.001
Bladder	1.41 (1.27-1.56)	<.001	1.81 (1.65-1.98)	<.001	1.09 (0.98-1.21)	.13	1.13 (1.03-1.24)	.009
Esophagus	3.17 (2.80-3.59)	<.001	0.66 (0.51-0.86)	.002	2.13 (1.88-2.41)	<.001	0.55 (0.42-0.71)	<.001
Leukemia	0.83 (0.72-0.95)	.008	0.86 (0.74-1.00)	.05	0.73 (0.63-0.85)	<.001	1.49 (1.25-1.77)	<.001
Brain and other nervous system	1.12 (0.96-1.31)	.15	0.58 (0.46-0.73)	<.001	1.38 (1.18-1.61)	<.001	0.41 (0.33-0.52)	<.001
Stomach	1.85 (1.62-2.11)	<.001	0.52 (0.39-0.68)	<.001	1.70 (1.48-1.94)	<.001	0.58 (0.44-0.76)	<.001
Other	1.40 (1.29-1.52)	<.001	1.07 (0.98-1.17)	.14	1.34 (1.23-1.46)	<.001	0.98 (0.89-1.07)	.58

Abbreviations: HR, hazard ratio; NA, not applicable.

^a^ Cox proportional hazards regression models controlled for other cause of death as competing risks. Time interaction terms were included for factors for which proportional hazards assumption was not met. Models also adjusted for characteristics in Table 2 and age at diagnosis, North American Association of Central Cancer Registries, and year of diagnosis, with results shown in the eFigure in Supplement 1.

^b^ Male breast cancer was grouped into the “other” category.

## Discussion

This cohort study provides national estimates of suicide risks associated with cancer and identifies risk factors of suicide among individuals diagnosed with cancer using data from population-based cancer registries of 43 states in 2000 to 2016 in the US. We found that the elevated suicide risk associated with cancer decreased during the study period, coinciding with increased use of psychosocial and palliative care and advances in symptom management.^21,22,23^ However, the suicide risk among individuals with cancer remained higher than the general population in all years. Geographic, racial and ethnic, socioeconomic, and clinical characteristics, some of which are modifiable, contributed to the elevated suicide risks. The highest suicide risk occurred in the first 6 months after diagnosis, during which individuals diagnosed with cancer bore more than 7 times the suicide risk of the general population. Cox proportional hazards regression analysis among individuals diagnosed with cancer showed that older age, distant stage, and cancer types with poor prognosis and high symptom burden^24^ (eg, cancers of the oral cavity and pharynx, esophagus, stomach, brain and other nervous system, pancreas, and lung and bronchus) had higher risks of suicide in the first 2 years of diagnosis. After 2 years, individuals with oral cavity and pharynx cancers and other cancer types subject to long-term quality-of-life impairment (eg, cancers of female breast, uterine and bladder, and leukemia) had higher suicide risks. These findings can inform clinical practice and treatment guidelines to better address patients’ needs for psychosocial supports and symptom management, including palliative care.

Both the SMR estimates from comparisons with the general population and the HR estimates from comparisons only among individuals diagnosed with cancer showed a decreasing trend of suicide risks during 2000 to 2016. Our analysis using a defined cancer cohort confirms a previous finding based on death certificate data, which reported decreasing cancer-related suicide rates in the past 2 decades.^25^ Moreover, our data supplement death certificates with richer information on key socially and clinically relevant characteristics, including health insurance type and follow-up time. These decreasing trends coincide with the greater incorporation of psychosocial and palliative care into oncologic care.^21,22,23^ Similarly, continuous efforts in education and training of oncologists, psycho-oncologists, and palliative care specialists and development of clinical guidelines have been put into place in the past decades.^26,27,28^

To our knowledge, this is the first time the NAACCR CiNA data, which comprise the largest cohort of individuals with newly diagnosed cancer in the US, have been used to examine suicide risk. We filled critical gaps in the literature besides confirming the risk factors identified by previous studies (eg, male, older age, and living in poor areas) in limited geographic areas.^3,4,5^ First, with wider geographic areas, we identified several states with high suicide risks—Alaska, Nevada, and New Mexico—some of which were non-SEER states and not previously studied. These results may be related to the suicide clusters among American Indian and Alaska Native communities.^29^ The political, social, cultural, and economic environment may also contribute to the suicide risk in an area and merit further research.^30^ For example, Medicaid expansion under the Patient Protection and Affordable Care Act was recently found to be associated with decreased suicide rates among both the general population and individuals diagnosed with cancer.^31,32^ Second, Hispanic and American Indian, Alaska Native, Asian, and Pacific Islander individuals with cancer were at substantially higher suicide risks than their peers without cancer, which points to potential barriers to health care resources, structural racism, and difficulties navigating health care systems among these populations.^33,34^ This finding underscores the importance of increasing diversity, language, and cultural competence among health care professionals, improving health insurance coverage, and tailoring psychosocial support for Hispanic and American Indian, Alaska Native, Asian, and Pacific Islander individuals diagnosed with cancer. Third, we characterized suicide risks associated with cancer by insurance type. Besides the uninsured, Medicare beneficiaries 64 years or younger (whose eligibility for coverage is based on certain medical conditions or disability), Medicaid-insured individuals (whose eligibility for coverage is mostly based on low-income),^35,36^ and individuals with VA or IHS/PHS coverage (2 populations at increased risks of mental health conditions and suicide),^37,38^ all had substantially higher suicide risks than the general population. Hence, expanding insurance coverage to the uninsured population and ensuring comprehensive coverage for cancer care and mental health services, including suicide screening and prevention as well as symptom management and palliative care by all Medicare, Medicaid, VA, and IHS/PHS programs, are crucial.

Our in-depth analyses showed that suicide risks associated with stage and cancer type are time dependent. During the first 2 years after the cancer diagnosis, when most individuals undergo active treatments, late-stage cancer and cancer types with poor prognosis and heavy symptom burdens had the highest suicide risks (eg, cancers of the lung and bronchus, oral cavity and pharynx, esophagus, pancreas, and stomach), which is consistent with prior research.^3,5,9^ After 2 years, when many individuals transition to survivorship care, those with cancer types that are typically subject to physical and psychological long-term and late effects, functional impairments, and poor quality of life (eg, female breast cancer, uterine cancer, bladder cancer, and leukemia) had higher suicide risks. These findings have implications for oncologic care in both early and later survivorship phases. Suicide screening and prevention should be prioritized among individuals recently diagnosed with fatal cancers and be incorporated into long-term survivorship care. Considering the dynamic pattern of care delivery with time since cancer diagnosis, our findings also point to the importance of multilevel interventions from federal and state government–level health care reforms to ensure adequate access to care to practitioner-level engagement, including oncologists, psycho-oncologists, primary care physicians, mental health professionals, and social workers, throughout the cancer care continuum.

Our estimated SMRs were smaller than those in previous studies^3,5,7^ overall and for different subgroups. This finding could be due to our inclusion of more recent data and decreasing trends of suicide among individuals with cancer, better representation of individuals with cancer in the US (eg, we included Maryland and Rode Island, which had relatively low suicide rates and were not included in previous SEER estimates), and improvement in risk standardization using attained age at death. Many previous studies^3,5,39,40^ used age at diagnosis among the cancer cohort but attained age at death among the general population for age standardization. This approach could lead to biased estimates because suicide could occur years after cancer diagnosis given the improvement in cancer survival in the past decades^41^ and the fact that suicide rates vary substantially by age.^42,43^ Another advantage of our study is that we used robust analytic models to estimate risk factors among the cancer cohort, such as including other causes of death as competing risks and time-varying interaction terms that allowed for different hazards in the period immediately after diagnosis and long-term survivorship.

### Limitations

Our study has several limitations. First, exact attained age at death and age at diagnosis were not available and were imputed based on 5-year age intervals. However, our sensitivity analyses using alternative imputation methods (at the minimum and maximum age in the 5-year age intervals) showed similar results (eTable 2 in Supplement 1). Second, the availability of health insurance coverage information varied by state and year (eTable 1 in Supplement 1). However, no systematic patterns were observed, and the direction of our estimates by insurance type was as expected. Our estimations by insurance type should be calibrated with more complete data in future studies as the quality of payer data improves.^44^ Third, we could not estimate differential risks by treatment because of the lack of detailed treatment information. Cancer treatments combined with supportive care can improve prognosis while reducing adverse effects. Examining the associations between cancer treatment(s), including supportive care, and suicide risk is an important area for future research. Fourth, our data did not include selected cancer registries that declined participation or data being unusable for survival analysis. However, cancer incident cases from the 43 states of our study represent a greater than 80% cancer incidence in the nation.^45^ Fifth, the cause of death from death certificates may be subject to reporting bias.^46^ However, we do not expect such bias to differ between the general population and individuals with cancer.

## Conclusions

In this cohort study of individuals diagnosed with cancer from 43 US states during the past 2 decades, we found that suicide risks decreased among individuals with cancer but remained higher compared with the general population. Geographic, racial and ethnic, socioeconomic, and clinical characteristics, some of which are modifiable, contributed to the elevated suicide risks among individuals diagnosed with cancer. Screening and tailored social and psycho-oncologic interventions are needed for suicide prevention in this vulnerable population. These interventions require joint efforts by federal and state governments, as well as health care institutions and practitioners, to ensure comprehensive health insurance coverage for psycho-oncologic, psychosocial, and palliative care; development of appropriate clinical guidelines for suicide risk screening; and inclusion of suicide prevention in survivorship care plans.

## Notes

Supplement 1. **eTable 1.** Percent of Individuals Missing Insurance Information by State and Year of Cancer Diagnosis **eTable 2.** Standard Mortality Ratios Using Alternative Imputation Approaches **eTable 3.** Risk of Suicide Associated with Individuals’ Characteristics Among Cancer Cohort, 2005-2016 **eFigure.** Adjusted Hazard Ratios from Multivariable Cox Proportional Hazards Model

Supplement 2.
Data Sharing Statement
