# Supplementary material for: Suicide Risk Among Individuals Diagnosed With Cancer in the US, 2000-2016
Source: JAMA Netw Open. 2023 Jan 20;6(1):e2251863. doi: 10.1001/jamanetworkopen.2022.51863 (PMC9860529; doi:10.1001/jamanetworkopen.2022.51863)
Supplement: Supplement 1. — eTable 1. Percent of Individuals Missing Insurance Information by State and Year of Cancer Diagnosis eTable 2. Standard Mortality Ratios Using Alternative Imputation Approaches eTable 3. Risk of Suicide Associated with Individuals’ Characteristics Among Cancer Cohort, 2005-2016 eFigure. Adjusted Hazard Ratios from Multivariable Cox Proportional Hazards Model [file jamanetwopen-e2251863-s001.pdf]

Supplementary Online Content

Hu X, Ma J, Jemal A, et al. Suicide risk among individuals diagnosed with cancer in the US, 2000-2016. *JAMA Netw Open*. 2023;6(1):e2251863. doi:10.1001/jamanetworkopen.2022.51863

eTable 1. Percent of Individuals Missing Insurance Information by State and Year of Cancer Diagnosis
eTable 2. Standard Mortality Ratios Using Alternative Imputation Approaches
eTable 3. Risk of Suicide Associated With Individuals' Characteristics among Cancer Cohort, 2005-2016
eFigure. Adjusted Hazard Ratios from Cox Proportional Hazards Model

This supplementary material has been provided by the authors to give readers additional information about their work.

© 2023 Hu X et al. *JAMA Network Open.*

eTable 1. Percent of Individuals Missing Insurance Information by State and Year of Cancer Diagnosis

State	Year																
	2000	2001	2002	2003	2004	2005	2006	2007	2008	2009	2010	2011	2012	2013	2014	2015	2016
Alabama	15.4%	11.3%	8.5%	8.3%	8.8%	10.8%	10.0%	8.5%	10.0%	9.1%	10.8%	11.7%	13.5%	11.1%	8.1%	9.9%	11.0%
Alaska	52.8%	39.9%	26.3%	26.1%	25.4%	28.5%	26.8%	8.9%	7.4%	10.0%	17.2%	16.3%	11.8%	26.1%	27.7%	29.3%	16.3%
Arizona	9.0%	8.6%	10.9%	14.2%	13.7%	13.4%	12.3%	7.6%	7.3%	9.7%	10.6%	9.6%	10.3%	9.2%	10.2%	10.5%	11.3%
Arkansas	3.7%	4.2%	7.0%	11.5%	8.9%	8.1%	8.2%	3.8%	9.4%	10.7%	8.4%	5.6%	6.9%	8.4%	6.3%	4.0%	4.0%
California	4.1%	3.4%	3.5%	3.8%	3.4%	3.5%	4.0%	100.0%	100.0%	100.0%	100.0%	100.0%	100.0%	100.0%	100.0%	100.0%	100.0%
Colorado	14.9%	12.7%	10.4%	11.0%	11.6%	10.0%	12.6%	12.1%	12.9%	11.4%	11.0%	9.5%	8.5%	7.9%	8.1%	5.4%	5.9%
Connecticut	100.0%	100.0%	100.0%	100.0%	100.0%	100.0%	100.0%	8.7%	5.9%	6.3%	5.0%	4.7%	4.7%	4.8%	4.5%	3.3%	2.7%
Delaware	13.0%	14.9%	14.2%	13.3%	10.7%	12.3%	7.7%	8.5%	9.0%	10.4%	12.4%	12.5%	13.5%	11.9%	9.9%	11.5%	10.3%
District of Columbia	9.3%	8.3%	12.5%	16.5%	7.1%	7.5%	11.5%	9.2%	6.9%	9.4%	6.1%	10.4%	11.2%	6.2%	7.7%	10.4%	5.9%
Florida	4.3%	4.1%	3.8%	3.5%	3.1%	3.2%	3.3%	3.2%	3.4%	3.3%	4.0%	6.1%	6.2%	8.0%	7.3%	8.0%	8.0%
Georgia	99.6%	99.6%	99.5%	99.5%	98.9%	98.2%	7.4%	6.4%	5.9%	5.8%	6.5%	7.5%	7.9%	7.4%	8.4%	8.7%	10.1%
Hawaii	100.0%	100.0%	100.0%	100.0%	100.0%	100.0%	100.0%	9.8%	11.4%	11.6%	12.8%	18.0%	14.2%	14.0%	9.5%	9.0%	7.9%
Idaho	16.2%	16.5%	14.0%	12.5%	12.1%	10.3%	10.5%	7.7%	7.7%	8.5%	7.9%	5.9%	6.0%	65.2%	41.6%	39.8%	38.9%
Iowa	100.0%	100.0%	100.0%	100.0%	100.0%	100.0%	100.0%	7.3%	6.7%	7.0%	7.1%	4.9%	3.5%	3.8%	3.7%	3.8%	3.9%
Kentucky	100.0%	100.0%	100.0%	100.0%	100.0%	100.0%	100.0%	4.6%	4.1%	4.6%	4.7%	4.9%	5.8%	5.3%	5.2%	4.5%	5.5%
Louisiana	100.0%	100.0%	100.0%	100.0%	100.0%	100.0%	100.0%	6.3%	4.6%	4.5%	4.2%	4.2%	5.6%	4.7%	4.3%	4.4%	3.5%
Maine	24.1%	10.0%	6.8%	6.2%	4.9%	5.0%	4.6%	5.4%	4.3%	4.6%	4.9%	4.8%	4.0%	3.6%	3.5%	3.1%	3.6%
Maryland	22.0%	23.6%	24.1%	16.4%	13.7%	14.0%	10.8%	7.5%	8.2%	8.5%	9.1%	7.1%	5.9%	5.4%	5.6%	5.8%	6.7%
Michigan	62.1%	59.3%	29.7%	17.1%	31.6%	37.9%	28.3%	23.8%	40.4%	41.5%	42.4%	42.6%	40.0%	41.3%	43.3%	42.8%	39.4%
Minnesota	100.0%	100.0%	100.0%	100.0%	100.0%	100.0%	100.0%	100.0%	100.0%	100.0%	100.0%	100.0%	100.0%	100.0%	100.0%	100.0%	100.0%
Mississippi	-	-	78.4%	12.0%	10.1%	11.6%	9.8%	6.2%	3.7%	4.3%	5.1%	3.2%	4.3%	4.8%	3.9%	2.8%	2.3%
Missouri	98.5%	97.8%	97.9%	97.2%	94.2%	87.5%	60.9%	6.3%	5.0%	5.0%	5.6%	6.0%	4.8%	2.5%	2.2%	2.3%	2.4%
Montana	18.6%	16.4%	13.8%	13.3%	11.0%	8.4%	8.4%	6.2%	5.7%	5.2%	4.0%	3.8%	5.4%	4.6%	3.8%	4.9%	3.6%
Nebraska	14.3%	12.1%	12.4%	11.8%	9.4%	10.2%	8.8%	8.8%	8.4%	8.5%	9.8%	8.2%	7.9%	8.8%	8.4%	8.2%	11.3%
Nevada	55.6%	58.9%	61.3%	49.8%	35.8%	28.2%	21.6%	17.6%	21.0%	21.6%	18.0%	20.4%	19.9%	16.9%	18.1%	14.6%	15.1%
New Hampshire	17.5%	13.0%	11.9%	10.7%	6.3%	7.4%	5.3%	6.2%	4.6%	4.8%	5.0%	4.6%	4.1%	3.7%	4.0%	4.9%	2.1%
New Jersey	100.0%	100.0%	100.0%	100.0%	100.0%	100.0%	100.0%	11.1%	11.2%	10.6%	11.5%	10.5%	11.1%	9.7%	9.3%	9.6%	8.6%
New Mexico	100.0%	100.0%	100.0%	100.0%	100.0%	100.0%	100.0%	14.3%	12.1%	12.8%	12.5%	9.7%	8.9%	8.1%	14.0%	12.5%	14.0%
North Carolina	93.4%	79.9%	22.6%	9.9%	6.4%	5.4%	4.0%	4.4%	4.4%	5.5%	4.8%	4.4%	5.1%	6.5%	5.6%	4.9%	4.3%

© 2023 Hu X et al. *JAMA Network Open.*

North Dakota	60.9%	20.9%	17.6%	9.7%	7.0%	5.3%	5.6%	4.7%	5.1%	4.3%	5.5%	3.5%	3.5%	6.0%	4.9%	4.1%	5.7%
Ohio	16.6%	16.9%	15.5%	18.0%	15.3%	11.1%	9.9%	9.8%	11.1%	7.7%	4.1%	3.5%	3.4%	2.9%	3.1%	2.5%	2.3%
Oklahoma	5.5%	4.4%	3.8%	4.0%	3.6%	4.5%	4.2%	5.4%	9.8%	11.1%	9.9%	11.6%	13.9%	11.9%	13.2%	8.3%	6.9%
Oregon	69.6%	14.3%	16.2%	13.5%	13.5%	13.5%	11.3%	10.4%	10.2%	10.7%	10.3%	12.1%	8.9%	8.7%	7.2%	7.9%	5.5%
Pennsylvania	97.3%	96.7%	96.0%	94.3%	89.7%	59.3%	3.5%	3.0%	2.6%	2.6%	3.2%	2.6%	2.5%	2.2%	1.4%	1.8%	1.6%
Rhode Island	8.9%	6.9%	7.4%	6.5%	5.2%	5.0%	6.1%	5.3%	5.4%	6.9%	6.5%	6.2%	5.7%	5.0%	4.8%	3.0%	1.1%
Tennessee	17.0%	15.6%	14.7%	15.3%	14.3%	22.2%	12.5%	6.3%	6.3%	6.3%	5.4%	5.7%	5.8%	5.6%	5.5%	6.3%	5.3%
Texas	98.9%	98.8%	98.6%	98.4%	97.5%	94.2%	64.9%	10.0%	9.6%	11.4%	10.8%	11.5%	10.3%	10.1%	10.0%	9.1%	6.0%
Utah	100.0%	100.0%	100.0%	100.0%	100.0%	100.0%	100.0%	11.2%	24.0%	16.5%	11.8%	15.3%	13.4%	13.1%	12.2%	14.6%	15.2%
Virginia	13.7%	15.6%	17.2%	16.5%	18.1%	22.8%	18.9%	13.5%	9.8%	9.4%	5.3%	6.4%	8.0%	8.0%	4.8%	4.1%	4.3%
Washington	99.2%	99.1%	97.7%	87.5%	79.9%	75.5%	74.0%	12.7%	12.1%	18.5%	8.9%	7.8%	7.8%	10.1%	14.4%	15.7%	6.3%
West Virginia	26.4%	23.1%	30.9%	26.7%	21.4%	12.3%	6.2%	6.5%	6.9%	5.7%	4.6%	3.8%	3.9%	2.7%	2.0%	2.4%	2.5%
Wisconsin	93.2%	91.9%	90.4%	82.9%	11.9%	13.6%	7.2%	4.2%	2.9%	2.0%	2.3%	2.6%	1.5%	1.2%	1.1%	1.4%	0.8%
Wyoming	48.3%	45.5%	49.1%	44.0%	31.7%	28.5%	36.0%	22.7%	20.0%	19.2%	23.8%	30.6%	30.2%	28.9%	26.1%	23.0%	22.2%

Notes: Cancer cases from Mississippi were not reported in North American Association of Central Cancer Registries program in 2000-2001.

© 2023 Hu X et al. *JAMA Network Open.*

eTable 2. Standard Mortality Ratios Using Alternative Imputation Approaches

	Imputation using minimum age within 5-year age intervals			Imputation using maximum age within 5-year age intervals	
	SMR[a]	95% CI		SMR[a]	95% CI
**Overall**	1.28	(1.26-1.30)		1.24	(1.22-1.25)
**Year of Death**					
**2000**	1.67	(1.47-1.88)		1.67	(1.47-1.88)
**2001**	1.65	(1.50-1.80)		1.58	(1.44-1.73)
**2002**	1.53	(1.40-1.65)		1.44	(1.33-1.56)
**2003**	1.51	(1.40-1.62)		1.43	(1.32-1.53)
**2004**	1.41	(1.31-1.50)		1.34	(1.24-1.43)
**2005**	1.33	(1.25-1.42)		1.27	(1.19-1.36)
**2006**	1.26	(1.18-1.34)		1.22	(1.14-1.29)
**2007**	1.33	(1.25-1.41)		1.29	(1.21-1.36)
**2008**	1.19	(1.12-1.26)		1.16	(1.10-1.23)
**2009**	1.30	(1.23-1.37)		1.27	(1.21-1.34)
**2010**	1.16	(1.10-1.22)		1.14	(1.08-1.20)
**2011**	1.21	(1.15-1.27)		1.19	(1.13-1.25)
**2012**	1.14	(1.08-1.20)		1.12	(1.06-1.18)
**2013**	1.20	(1.15-1.26)		1.17	(1.12-1.23)
**2014**	1.19	(1.13-1.24)		1.16	(1.11-1.21)
**2015**	1.22	(1.17-1.28)		1.20	(1.15-1.25)
**2016**	1.17	(1.12-1.22)		1.15	(1.10-1.20)
**Alive**	NA	NA		NA	NA
**Attained age at death**					
**0-24 years**	0.93	(0.76-1.11)		1.23	(0.99-1.46)
**25-39 years**	0.95	(0.87-1.03)		1.24	(1.13-1.34)
**40-49 years**	0.95	(0.90-1.00)		1.33	(1.25-1.40)
**50-54 years**	0.99	(0.94-1.04)		1.37	(1.30-1.44)
**55-59 years**	1.06	(1.01-1.10)		1.34	(1.28-1.40)
**60-64 years**	1.23	(1.18-1.28)		1.43	(1.38-1.49)
**65-69 years**	1.41	(1.35-1.46)		1.51	(1.45-1.56)
**70-74 years**	1.42	(1.37-1.47)		1.34	(1.29-1.39)
**75-79 years**	1.49	(1.44-1.55)		1.28	(1.23-1.32)
**80-84 years**	1.48	(1.42-1.54)		1.07	(1.03-1.12)
**85+ years**	1.55	(1.48-1.62)		0.75	(0.72-0.79)
**Sex**					
**Male**	1.32	(1.30-1.34)		1.25	(1.23-1.26)
**Female**	1.11	(1.07-1.15)		1.19	(1.15-1.23)
**Race/Ethnicity**					
**Hispanic**	1.52	(1.41-1.62)		1.43	(1.33-1.53)
**Non-Hispanic AIANAPI**	1.82	(1.66-1.98)		1.75	(1.59-1.90)
**Non-Hispanic Black**	1.15	(1.06-1.24)		1.15	(1.06-1.24)
**Non-Hispanic White**	1.27	(1.25-1.28)		1.22	(1.21-1.24)
**Region**					
**Northeast**	1.25	(1.20-1.31)		1.24	(1.19-1.30)
**Midwest**	1.27	(1.23-1.31)		1.24	(1.20-1.28)
**South**	1.23	(1.20-1.25)		1.18	(1.16-1.21)
**West**	1.21	(1.18-1.24)		1.15	(1.12-1.18)
**Insurance type[b]**					
**Private**	1.07	(1.04-1.10)		1.08	(1.05-1.11)
**Medicaid-no Medicare**	1.75	(1.63-1.86)		1.70	(1.59-1.82)
**Medicare ≤64 y of age**	1.91	(1.77-2.04)		1.98	(1.84-2.11)
**Medicare ≥65 y of age**	1.48	(1.44-1.52)		1.35	(1.31-1.38)
**VA or IHS/PHS**	1.92	(1.75-2.08)		1.85	(1.69-2.01)
**Uninsured**	1.66	(1.53-1.80)		1.67	(1.53-1.80)

© 2023 Hu X et al. *JAMA Network Open.*

**Unknown or missing**	1.19	(1.16-1.22)		1.14	(1.12-1.17)
**County-level Rurality[c]**					
**Metropolitan**	1.30	(1.27-1.32)		1.28	(1.25-1.30)
**Non-Metropolitan Urban**	1.36	(1.30-1.41)		1.32	(1.26-1.37)
**Non-Metropolitan Rural**	1.41	(1.26-1.56)		1.36	(1.21-1.51)
**County-level poverty, %[c]**					
**< 5.0**	1.24	(1.13-1.35)		1.23	(1.12-1.33)
**5.0 - 9.99**	1.43	(1.38-1.48)		1.41	(1.37-1.46)
**10.0 - 19.99**	1.36	(1.33-1.39)		1.33	(1.30-1.36)
**≥20.0**	1.36	(1.27-1.46)		1.34	(1.25-1.43)
**Stage**					
**In situ or local**	1.06	(1.04-1.08)		1.02	(1.00-1.04)
**Regional**	1.47	(1.42-1.51)		1.44	(1.40-1.49)
**Distant**	1.91	(1.85-1.97)		1.87	(1.81-1.93)
**Not available, unknown, or missing**	1.65	(1.57-1.72)		1.58	(1.51-1.65)
**Multiple Primary**					
**1**	1.37	(1.35-1.39)		1.33	(1.31-1.35)
**≥2**	0.81	(0.77-0.84)		0.77	(0.74-0.80)
**Time since Diagnosis at Death**					
**0-5 mo**	7.20	(6.98-7.42)		7.13	(6.92-7.35)
**6-11 mo**	5.61	(5.37-5.86)		5.49	(5.25-5.73)
**12-23 mo**	4.20	(4.05-4.36)		4.10	(3.95-4.25)
**24-35 mo**	3.12	(2.99-3.25)		3.02	(2.89-3.14)
**3-4 y**	2.01	(1.94-2.09)		1.94	(1.87-2.01)
**5-9 y**	0.96	(0.93-0.98)		0.92	(0.90-0.95)
**≥10 y**	0.24	(0.23-0.25)		0.24	(0.22-0.25)
**Primary Site**					
**Female breast[e]**	0.99	(0.93-1.05)		1.07	(1.01-1.13)
**Prostate**	0.94	(0.91-0.96)		0.88	(0.85-0.90)
**Colon and rectum**	1.27	(1.22-1.33)		1.23	(1.18-1.28)
**Lung and bronchus**	2.36	(2.27-2.46)		2.30	(2.21-2.39)
**Uterine corpus**	1.08	(0.98-1.18)		1.16	(1.05-1.27)
**Oral cavity and pharynx**	2.43	(2.29-2.57)		2.41	(2.27-2.55)
**Kidney and renal pelvis**	1.15	(1.06-1.24)		1.13	(1.04-1.21)
**Melanoma**	1.04	(0.98-1.10)		1.01	(0.95-1.07)
**Non-Hodgkin lymphoma**	1.19	(1.11-1.27)		1.15	(1.08-1.23)
**Thyroid**	0.82	(0.73-0.92)		0.82	(0.73-0.91)
**Pancreas**	2.51	(2.25-2.77)		2.47	(2.21-2.73)
**Liver and intrahepatic bile duct**	1.59	(1.36-1.82)		1.58	(1.36-1.81)
**Bladder**	1.31	(1.24-1.37)		1.23	(1.16-1.29)
**Esophagus**	3.18	(2.85-3.50)		3.08	(2.77-3.40)
**Leukemia**	1.19	(1.09-1.30)		1.14	(1.04-1.24)
**Brain and other nervous system**	1.71	(1.50-1.92)		1.63	(1.43-1.84)
**Stomach**	2.35	(2.09-2.61)		2.28	(2.03-2.53)
**Other**	1.64	(1.57-1.70)		1.59	(1.52-1.65)

Abbreviations: AIANAPI, American Indian, Alaska Native, Asian, or Pacific Islander; IHS, Indian Health Service; NA, not applicable; PHS, Public Health Service; SMR, standardized mortality ratio; VA, Veterans Affairs.

[a] The SMRs were standardized by attained age at death, sex, and race and ethnicity. The SMRs for year of death, region, county-level rurality, and county-level poverty subcategories were also standardized by year of death, region, county-level rurality, and county-level poverty, respectively. Imputation for exact age at cancer diagnosis was assumed as the minimum or the maximum value of the corresponding age group.

[b] Private insurance included private fee-for-service and managed care, TRICARE, and military insurance; Medicaid included traditional Medicaid, managed care Medicaid, and other not specified Medicaid without Medicare; Medicare included traditional fee-for-service Medicare, Medicare administered through a managed care plan, Medicare with supplemental coverage, and Medicare with Medicaid eligibility.

[c] County-level data only available in 2005 and after (N= 11 867 171). County-level rurality were coded with Rural-Urban Continuum Codes developed by the United States Department of Agriculture (https://seer.cancer.gov/seerstat/variables/countyattribs/ruralurban.html). County-level poverty were coded with percent of persons below poverty data from the American Community Survey 2007-2011 (https://seer.cancer.gov/seerstat/variables/countyattribs/static.html#07-11).

[e] Male breast cancer was grouped into the “Other” category.

© 2023 Hu X et al. *JAMA Network Open.*

eTable 3. Risk of Suicide Associated with Individuals' Characteristics among Cancer Cohort, 2005-2016

	**Univariable Regression (n=11,867,171)**				**Multivariable Regression (n=11,867,171)**			
**No time-interaction characteristics**	**HR (95% CI)**	**P-value**			**HR (95% CI)**	**P-value**		
Sex								
Female	Ref				Ref			
Male	5.01 (4.77-5.26)	<.001			5.44 (5.06-5.86)	<.001		
Race/Ethnicity								
Hispanic	0.56 (0.50-0.63)	<.001			0.57 (0.51-0.65)	<.001		
Non-Hispanic AIANAPI[a]	0.25 (0.23-0.28)	<.001			0.25 (0.23-0.28)	<.001		
Non-Hispanic Black	0.44 (0.41-0.49)	<.001			0.42 (0.38-0.47)	<.001		
Non-Hispanic White	Ref				Ref			
Insurance type[b]								
Private	Ref				Ref			
Medicaid	0.97 (0.89-1.07)	.54			1.21 (1.09-1.33)	<.001		
Medicare ≤64 years old	1.48 (1.35-1.61)	<.001			1.52 (1.39-1.66)	<.001		
Medicare 65+ years old	1.21 (1.16-1.27)	<.001			1.19 (1.11-1.26)	<.001		
VA or IHS/PHS	2.13 (1.91-2.37)	<.001			1.41 (1.26-1.58)	<.001		
Uninsured	1.24 (1.12-1.38)	<.001			1.32 (1.19-1.47)	<.001		
Unknown/Missing	1.12 (1.07-1.18)	<.001			1.06 (1.00-1.13)	.06		
State of residence								
California	Ref				Ref			
Alabama	1.01 (0.89-1.15)	.86			0.85 (0.74-0.98)	.02		
Alaska	1.60 (1.19-2.13)	.002			1.30 (0.98-1.72)	.074		
Arizona	1.15 (1.02-1.30)	.019			0.97 (0.86-1.10)	.64		
Arkansas	1.10 (0.94-1.28)	.23			0.83 (0.70-0.97)	.02		
Colorado	1.40 (1.24-1.59)	<.001			1.19 (1.05-1.36)	.008		
Connecticut	0.58 (0.48-0.70)	<.001			0.52 (0.42-0.63)	<.001		
Delaware	0.75 (0.54-1.02)	.066			0.66 (0.48-0.90)	.008		
District of Columbia	0.30 (0.15-0.57)	<.001			0.48 (0.23-1.00)	.05		
Florida	1.17 (1.08-1.26)	<.001			1.00 (0.92-1.09)	.96		
Georgia	1.02 (0.92-1.14)	.66			0.95 (0.85-1.06)	.39		
Hawaii	0.85 (0.66-1.11)	.24			0.94 (0.71-1.25)	.68		
Idaho	1.44 (1.18-1.75)	<.001			1.05 (0.86-1.29)	.62		
Iowa	0.86 (0.72-1.02)	.075			0.65 (0.54-0.77)	<.001		
Kentucky	1.07 (0.94-1.22)	.31			0.79 (0.69-0.91)	.001		
Louisiana	0.96 (0.83-1.10)	.52			0.88 (0.76-1.01)	.073		
Maine	0.95 (0.76-1.18)	.62			0.68 (0.54-0.85)	.001		
Maryland	0.63 (0.54-0.73)	<.001			0.65 (0.55-0.77)	<.001		
Michigan	0.85 (0.77-0.95)	.003			0.72 (0.65-0.81)	<.001		
Minnesota	NA				NA			
Mississippi	0.86 (0.73-1.03)	.097			0.76 (0.64-0.91)	.002		

© 2023 Hu X et al. *JAMA Network Open.*

Missouri	1.11 (0.99-1.25)	.067			0.90 (0.80-1.01)	.08		
Montana	1.51 (1.20-1.89)	<.001			1.04 (0.83-1.31)	.72		
Nebraska	0.94 (0.75-1.16)	.54			0.72 (0.58-0.90)	.003		
Nevada	1.88 (1.64-2.16)	<.001			1.57 (1.36-1.80)	<.001		
New Hampshire	0.93 (0.73-1.18)	.54			0.71 (0.56-0.91)	.006		
New Jersey	0.59 (0.52-0.67)	<.001			0.57 (0.50-0.65)	<.001		
New Mexico	1.58 (1.33-1.88)	<.001			1.46 (1.23-1.73)	<.001		
North Carolina	0.94 (0.85-1.04)	.25			0.81 (0.73-0.91)	<.001		
North Dakota	0.88 (0.62-1.24)	.45			0.61 (0.43-0.88)	.007		
Ohio	0.80 (0.72-0.88)	<.001			0.65 (0.58-0.73)	<.001		
Oklahoma	1.19 (1.04-1.37)	.012			0.94 (0.81-1.08)	.38		
Oregon	1.00 (0.86-1.16)	.98			0.79 (0.68-0.91)	.002		
Pennsylvania	0.89 (0.82-0.98)	.015			0.76 (0.69-0.84)	<.001		
Rhode Island	0.30 (0.19-0.48)	<.001			0.25 (0.15-0.43)	<.001		
Tennessee	1.08 (0.96-1.21)	.19			0.86 (0.76-0.97)	.01		
Texas	0.95 (0.88-1.03)	.22			0.90 (0.83-0.98)	.02		
Utah	1.36 (1.14-1.62)	.001			1.09 (0.91-1.31)	.33		
Virginia	0.91 (0.81-1.02)	.10			0.84 (0.74-0.95)	.005		
Washington	1.19 (1.06-1.33)	.002			0.97 (0.87-1.09)	.62		
West Virginia	1.12 (0.94-1.34)	.21			0.79 (0.66-0.95)	.013		
Wisconsin	0.91 (0.81-1.04)	.17			0.72 (0.63-0.83)	<.001		
Wyoming	0.16 (0.06-0.42)	<.001			0.11 (0.04-0.34)	<.001		
Year of diagnosis								
2005	Ref				Ref			
2006	1.00 (0.93-1.07)	.88			0.99 (0.92-1.07)	.82		
2007	1.02 (0.95-1.09)	.66			1.01 (0.93-1.09)	.79		
2008	0.92 (0.85-0.99)	.028			0.93 (0.86-1.01)	.08		
2009	0.99 (0.92-1.07)	.77			1.02 (0.94-1.10)	.65		
2010	0.87 (0.81-0.94)	.001			0.91 (0.84-0.99)	.02		
2011	0.83 (0.76-0.90)	<.001			0.87 (0.80-0.95)	.002		
2012	0.86 (0.80-0.94)	<.001			0.94 (0.86-1.02)	.16		
2013	0.83 (0.76-0.90)	<.001			0.91 (0.83-1.00)	.05		
2014	0.78 (0.71-0.85)	<.001			0.87 (0.79-0.96)	.005		
2015	0.73 (0.66-0.81)	<.001			0.83 (0.75-0.92)	<.001		
2016	0.66 (0.58-0.75)	<.001			0.73 (0.64-0.83)	<.001		
County-level Rurality[c]								
Metro	Ref				Ref			
NonMetro Urban	1.22 (1.16-1.27)	<.001			1.09 (1.03-1.14)	.001		
NonMetro Rural	1.31 (1.17-1.46)	<.001			1.14 (1.02-1.28)	.03		
County-level Poverty[c]								
< 5.0%	Ref				Ref			
5.0% - 9.99%	1.22 (1.11-1.34)	<.001			1.07 (0.97-1.18)	.17		

© 2023 Hu X et al. *JAMA Network Open.*

10.0% - 19.99%	1.33 (1.21-1.45)	<.001			1.13 (1.02-1.25)	.02		
20.0% +	1.17 (1.05-1.31)	.005			1.12 (0.99-1.27)	.07		
**Time-interacted characteristics**	Within 2 y		After 2 y		Within 2 y		After 2 y	
	**HR (95% CI)**	**P-value**	**HR (95% CI)**	**P-value**	**HR (95% CI)**	**P-value**	**HR (95% CI)**	**P-value**
Age at diagnosis								
0-24 years	Ref		Ref		Ref		Ref	
25-39 years	1.88 (1.30-2.71)	.001	1.31 (1.02-1.68)	.033	2.36 (1.63-3.42)	<.001	1.34 (1.05-1.72)	.02
40-49 years	2.65 (1.88-3.75)	<.001	1.43 (1.13-1.80)	.003	3.18 (2.24-4.51)	<.001	1.43 (1.13-1.81)	.003
50-54 years	3.03 (2.15-4.27)	<.001	1.34 (1.06-1.69)	.014	3.13 (2.20-4.44)	<.001	1.15 (0.91-1.46)	.25
55-59 years	3.17 (2.26-4.46)	<.001	1.27 (1.01-1.60)	.041	3.06 (2.16-4.33)	<.001	0.99 (0.78-1.26)	.94
60-64 years	3.34 (2.38-4.69)	<.001	1.22 (0.97-1.53)	.091	3.04 (2.15-4.30)	<.001	0.88 (0.69-1.11)	.28
65-69 years	3.69 (2.63-5.18)	<.001	1.29 (1.03-1.63)	.027	3.27 (2.31-4.62)	<.001	0.89 (0.70-1.13)	.32
70-74 years	4.03 (2.87-5.66)	<.001	1.30 (1.03-1.64)	.025	3.49 (2.46-4.94)	<.001	0.91 (0.72-1.16)	.44
75-79 years	4.80 (3.42-6.74)	<.001	1.20 (0.95-1.52)	.13	4.14 (2.93-5.87)	<.001	0.89 (0.69-1.13)	.33
80-84 years	4.24 (3.01-5.96)	<.001	1.00 (0.78-1.28)	.97	3.72 (2.62-5.29)	<.001	0.81 (0.63-1.04)	.10
85+ years	3.57 (2.53-5.04)	<.001	0.46 (0.33-0.64)	<.001	3.39 (2.38-4.82)	<.001	0.43 (0.30-0.60)	<.001
Stage								
In situ/Local	Ref		Ref		Ref		Ref	
Regional	1.44 (1.36-1.54)	<.001	0.74 (0.69-0.79)	<.001	1.32 (1.23-1.41)	<.001	0.88 (0.81-0.94)	<.001
Distant	1.62 (1.53-1.71)	<.001	0.39 (0.35-0.43)	<.001	1.31 (1.22-1.41)	<.001	0.47 (0.41-0.53)	<.001
NA/Unknown/Missing	1.83 (1.70-1.97)	<.001	0.57 (0.49-0.65)	<.001	1.43 (1.32-1.56)	<.001	0.67 (0.58-0.77)	<.001
Primary Site								
Colon and rectum	Ref		Ref		Ref		Ref	
Female breast[e]	0.28 (0.25-0.33)	<.001	0.45 (0.39-0.51)	<.001	1.05 (0.90-1.22)	.53	1.24 (1.07-1.44)	.004
Prostate	0.90 (0.82-1.00)	.040	1.80 (1.63-1.98)	<.001	0.62 (0.56-0.69)	<.001	0.99 (0.89-1.09)	.78
Lung and bronchus	1.45 (1.32-1.58)	<.001	0.44 (0.38-0.52)	<.001	1.24 (1.13-1.36)	<.001	0.54 (0.46-0.64)	<.001
Uterine corpus	0.29 (0.24-0.35)	<.001	0.37 (0.31-0.45)	<.001	1.07 (0.88-1.30)	.51	1.16 (0.94-1.43)	.18
Oral cavity and pharynx	2.58 (2.29-2.91)	<.001	2.27 (1.98-2.60)	<.001	1.94 (1.72-2.19)	<.001	1.60 (1.39-1.84)	<.001
Kidney and renal pelvis	0.98 (0.85-1.13)	.78	1.07 (0.91-1.26)	.42	0.96 (0.83-1.11)	.58	0.85 (0.72-1.00)	.05
Melanoma	0.90 (0.78-1.04)	.16	1.45 (1.27-1.66)	<.001	0.86 (0.74-1.00)	.05	0.99 (0.86-1.14)	.89
Non-Hodgkin lymphoma	0.97 (0.85-1.12)	.70	0.93 (0.79-1.09)	.37	0.92 (0.80-1.05)	.22	1.04 (0.88-1.22)	.64
Thyroid	0.40 (0.32-0.51)	<.001	0.76 (0.63-0.92)	.006	0.83 (0.66-1.04)	.11	0.94 (0.77-1.14)	.52
Pancreas	1.39 (1.21-1.59)	<.001	0.20 (0.11-0.37)	<.001	1.29 (1.12-1.49)	<.001	0.28 (0.15-0.51)	<.001
Liver and intrahepatic bile duct	0.91 (0.75-1.11)	.37	0.49 (0.33-0.72)	<.001	0.80 (0.65-0.97)	.02	0.41 (0.28-0.60)	<.001
Bladder	1.41 (1.25-1.59)	<.001	1.72 (1.51-1.95)	<.001	1.08 (0.95-1.22)	.23	1.09 (0.95-1.24)	.22
Esophagus	3.01 (2.60-3.50)	<.001	0.76 (0.54-1.06)	.11	1.96 (1.69-2.28)	<.001	0.61 (0.44-0.86)	.005
Leukemia	0.85 (0.72-1.00)	.049	0.87 (0.71-1.06)	.17	0.76 (0.64-0.90)	.002	1.39 (1.10-1.76)	.006
Brain and other nervous system	1.21 (1.01-1.45)	.038	0.51 (0.37-0.71)	<.001	1.48 (1.23-1.77)	<.001	0.39 (0.28-0.54)	<.001
Stomach	1.78 (1.52-2.09)	<.001	0.52 (0.35-0.75)	.001	1.64 (1.40-1.93)	<.001	0.57 (0.39-0.84)	.004
Other	1.37 (1.24-1.51)	<.001	1.02 (0.90-1.16)	.74	1.29 (1.17-1.43)	<.001	0.97 (0.86-1.10)	.65

© 2023 Hu X et al. *JAMA Network Open.*

Notes: Abbreviations: AIANAPI, American Indian, Alaska Native, Asian, or Pacific Islander; IHS, Indian Health Service; NA, not applicable; PHS, Public Health Service; SMR, standardized mortality ratio; VA, Veterans Affairs. Cox proportional hazard models controlled for other causes of death as competing risks. Time interaction terms were included for factors where proportional hazard assumption was not met.

[b] Private insurance included private fee-for-service and managed care, TRICARE, and military insurance; Medicaid included traditional Medicaid, managed care Medicaid, and other not specified Medicaid without Medicare; Medicare included traditional fee-for-service Medicare, Medicare administered through a managed care plan, Medicare with supplemental coverage, and Medicare with Medicaid eligibility.

[c] County-level data only available in 2005 and after (N= 11 867 171). County-level rurality were coded with Rural-Urban Continuum Codes developed by the United States Department of Agriculture (https://seer.cancer.gov/seerstat/variables/countyattribs/ruralurban.html). County-level poverty were coded with percent of persons below poverty data from the American Community Survey 2007-2011 (https://seer.cancer.gov/seerstat/variables/countyattribs/static.html#07-11).

[e] Male breast cancer was grouped into the “Other” category.

© 2023 Hu X et al. *JAMA Network Open.*

eFigure 1. Adjusted Hazard Ratios from Cox Proportional Hazards Model

A. By Year of Diagnosis

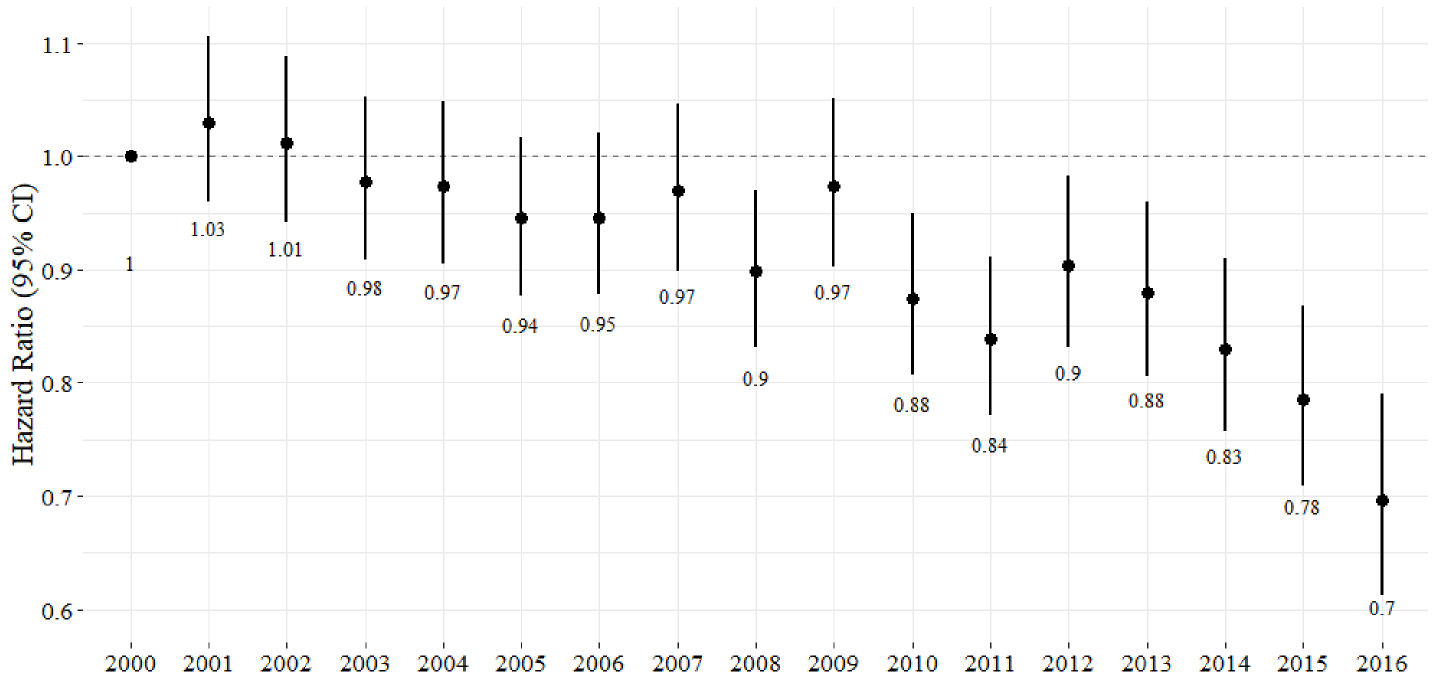

© 2023 Hu X et al. *JAMA Network Open.*

B. By State of Residence

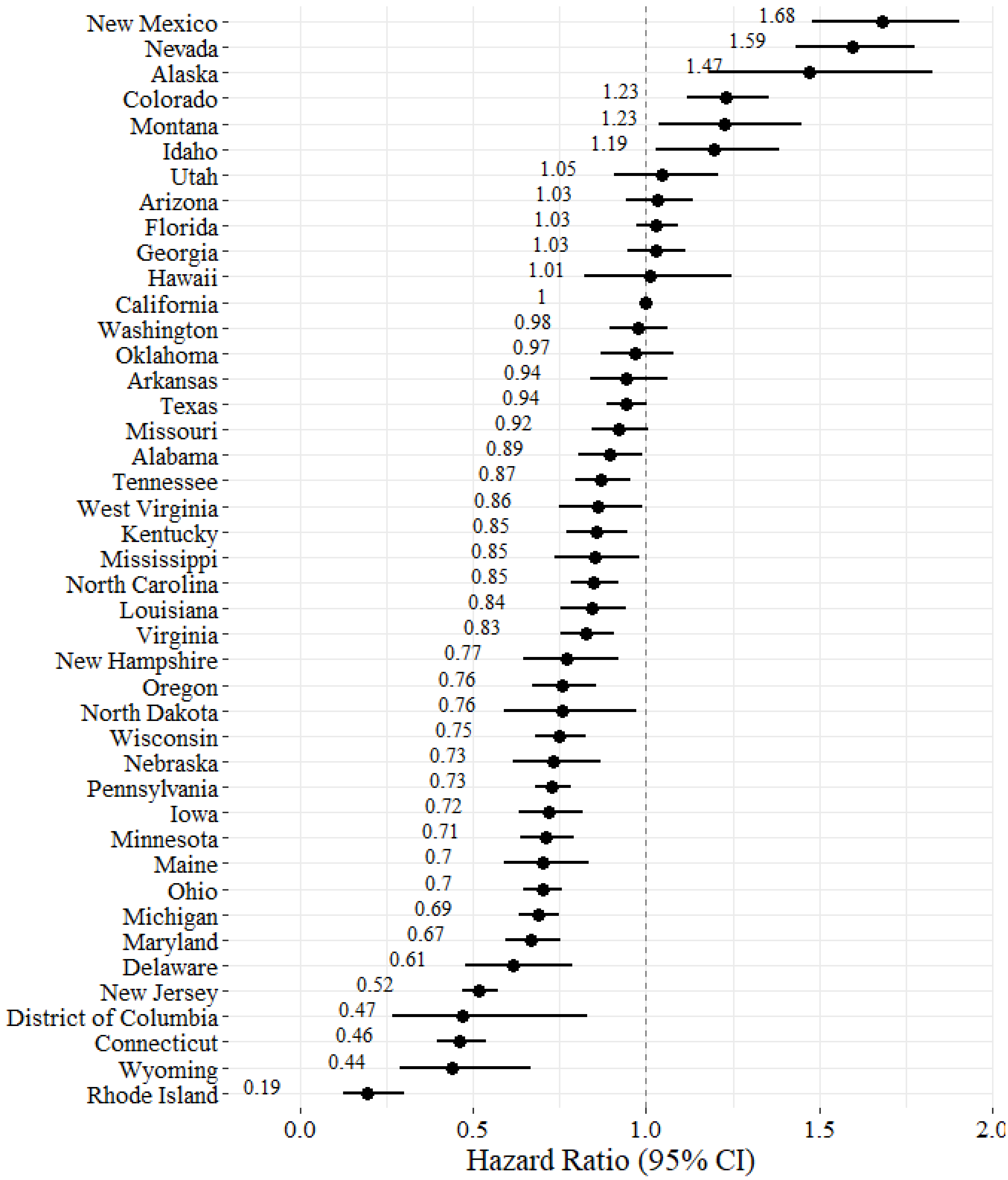

© 2023 Hu X et al. *JAMA Network Open.*

C. By Age at Diagnosis

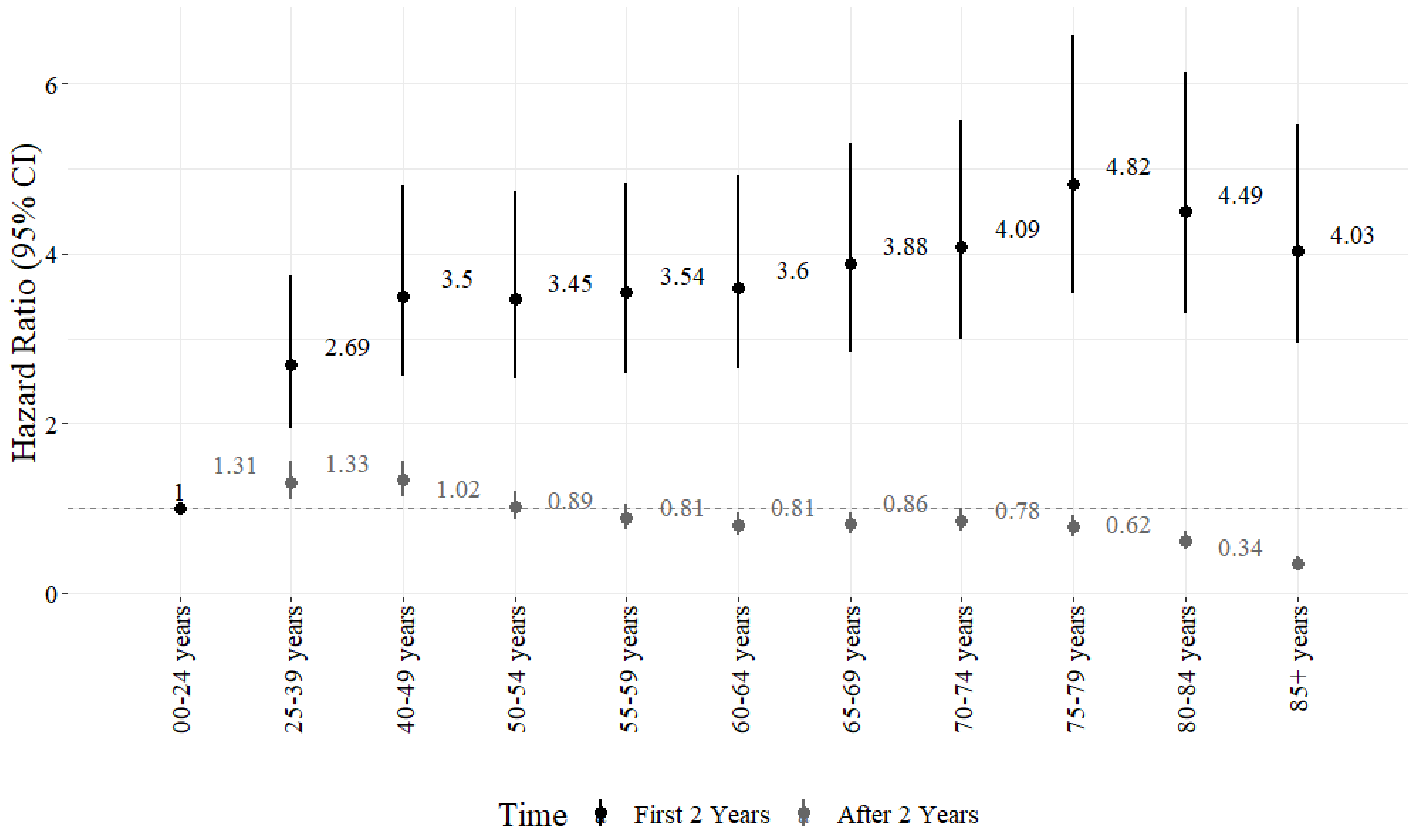

© 2023 Hu X et al. *JAMA Network Open.*
