# Supplementary material for: Suicide Risk Among Individuals Diagnosed With Cancer in the US, 2000-2016
Source: JAMA Netw Open. 2023 Jan 20;6(1):e2251863. doi: 10.1001/jamanetworkopen.2022.51863 (PMC9860529; doi:10.1001/jamanetworkopen.2022.51863)
Supplement: Supplement 2. — Data Sharing Statement [file jamanetwopen-e2251863-s002.pdf]

Data Sharing Statement

Hu. Suicide Risk Among Individuals Diagnosed With Cancer in the US, 2000-2016. *JAMA Netw Open*. Published January 20, 2023. doi:10.1001/jamanetworkopen.2022.51863

Data
Data available: No

Additional Information
Explanation for why data not available: The data underlying this article were provided by the American Association of Central Cancer Registries (NAACCR) by permission. The data cannot be shared publicly per the Data Use Agreement. The NAACCR CiNA Public Use Data Set with limited number of variables is available through application at https://www.naaccr.org/cina-public-use-data-set/.
